# Implementing dedicated education units in 6 European undergraduate nursing and midwifery students clinical placements

**DOI:** 10.1186/s12912-021-00576-5

**Published:** 2021-04-13

**Authors:** Sara Pedregosa, Núria Fabrellas, Ester Risco, Mariana Pereira, Małgorzata Stefaniak, Fisun Şenuzun, Sandra Martin, Adelaida Zabalegui

**Affiliations:** 1 Hospital Clinic de Barcelona, Barcelona, Spain; 2 Faculty of Medicine and Health Sciences, School of Nursing, University of Barcelona, Barcelona, Spain; 3 Parc Sanitari Pere Virgili, Barcelona, Spain; 4 Instituto Politécnico de Setúbal, Setúbal, Portugal; 5 Medical University of Warsaw, Warszawa, Poland; 6 Ege University Faculty of Nursing Internal Medicine Nursing, Izmir, Turkey; 7 Center of Expertise Health Innovation at UC Leuven-Limburg, Leuven, Belgium

**Keywords:** Dedicated education unit, Consolidated framework for implementation research, Clinical learning environment, Clinical education, Nursing students

## Abstract

**Background:**

Undergraduate students’ clinical experience, working directly with patients and the healthcare team is essential to ensure students acquire the necessary competence for practice. There are differences in the quality of clinical environments and in students’ clinical placement experiences and not all clinical sites are optimal learning environments. The Dedicated Education Unit clinical education model allows students to develop the practical knowledge, skills and professionalism they will need as nurses/midwives.

**Methods:**

We employed the Consolidated Framework for Implementation Research to identify and compare barriers and facilitators in the implementation of the Dedicated Education Unit in 6 European undergraduate nursing/midwifery student clinical placement settings and to describe the experience of nurses/midwives involved in the Dedicated Education Unit model implementation and evaluation. A pre-post implementation interpretive assessment was based on participants’ responses to the Consolidated Framework for Implementation Research construct questions.

**Results:**

Although Dedicated Education Unit model implementation in our project was heterogeneous, no main implementation barriers were perceived. Qualitative data showed that educational-service collaboration, including a focus on mutual goals, organizational communication and networking, satisfaction of educational and healthcare professionals, and the establishment of a safe space for professional discussion and feedback, were considered facilitators.

**Conclusions:**

This study describes the key elements guiding educational and healthcare stakeholders in Dedicated Education Unit implementation, engaging participants in the entire process, and offering other organizations the opportunity to consider the benefits of this clinical education model.

## Background

### European nursing educational context

The nursing and midwife learning process integrates theory, simulated practice and clinical placement (CP) into study programs. Although the main goal of the Bologna Declaration was to standardize European Higher Education, differences still exist in nursing curriculum implementation in Europe. There are both 3 and 4 year programs, inconsistent adoption of the European Credit Transfer System and wide variation in CP models and students’ experiences [1, 2]. As students’ CP represents more than 50% of nursing degree hours [3], it is vital to assist institutions and participants to deal with these issues.

### Clinical learning environment

The clinical learning environment includes the characteristics of the physical space, psychosocial factors, organizational culture, teaching and learning elements and everything that influences the student experience in achieving educational outcomes and developing knowledge and skills, behaviors and confidence [4, 5]. However, the literature describes differences in the quality of clinical environments and in students’ CP experiences, and shows that not all clinical sites are optimal learning environments which can, in some cases, have a negative impact on student learning [5, 6].

### Dedicated education units

The main goal of the Dedicated Education Unit (DEU) clinical education model is to create an optimal clinical learning environment through educational-service partnering. The DEU model allows nursing/midwifery students to achieve practical knowledge and skills and develop their professionalism and balances the demands of student learning and professionals roles, ensuring patient comfort and safety by improving quality of care. The DEU model strengthens the teaching role of the clinical nurse in instructing students’ clinical skills in the clinical setting. It also emphasizes the role of the faculty nurse in ensuring student knowledge acquisition and supporting the clinical nurses’ teaching role. In addition, the collaboration of educational and health institutions allows the optimization of training resources, enhances theory and practice integration, facilitates the student’ acquisition of competencies, and responds to professionals’ needs to provide appropriate nurse training [7–11]. Successful students and nurses’ outcomes in the DEU model are well documented [7–9].

The DEU model was implemented in the 1990s by nursing faculty from Flinders University in Australia. Then, in 2003, the University of Portland adapted the Australian DEU in an effort to improve the clinical learning environment and address a shortage of nurses. Since 2003, several educational and healthcare organizations in Australia, New Zealand, the United States of America and Europe have successfully implemented DEU units [7, 8, 10]. The essencial elements of DEU [11] are as follows:

- Committed partnership for education improvement between academic and healthcare organizations.
- Students’ CP duration should vary, depending on students’ curricula, from 6 to 12 weeks.
- A Clinical Mentor (CM) is an experienced and trained nurse/midwife who, in a one-on-one relationship, guides, instructs and supervises undergraduate students in clinical placement.
- A Link Teacher (LT) is an academic nurse/midwife hired by the Higher Education Institute without patient care responsibilities. As a part of a health team, the LT liaises between academic and healthcare organizations, responsible for clinical mentor-student partnership coordination and support, evaluation of the learning-teaching process, and student clinical placement assessment.
- The Head Nurse (HN) is the ward manager involved in the teaching-learning process, influencing staff motivation and creating the conditions for a ward learning culture.
- A mentoring course/program provides CM with pedagogical education, skills and support necessary to sustain students’ learning.
- Meetings between the LT, CM, HN and students are established to enhance feedback and communication, and agree on teaching/learning process planning, development and evaluation.

## Aims

The purposes of this study were (1) to identify and compare barriers and facilitators influencing the implementation of the DEU model in 6 European undergraduate nursing/midwifery student CP settings, and (2) to describe the experience of nursing/midwifery project coordinators involved in DEU implementation and evaluation.

## Method

### Study design

A multi-center qualitative study carried out in five European countries to identify facilitators, barriers and factors associated with DEU implementation in 6 European undergraduate nursing/midwifery students CP settings.

### Settings and participants

An innovative academic-service partnership between 6 European academic organizations and 6 European health care institutes in 5 European countries (Belgium, Portugal, Poland, Spain, Turkey) was established. University Colleges Leuven collaborated with University Hospital Leuven, University Colleges Limburg with Oost-Limburg Hospital, Medical University, Warsaw with Holy Family Hospital, Polytechnic Institute, Setúbal with Centro Hospitalar de Setúbal, Escola Infermeria de la Facultat de Medicina i Ciències de la Salut de la Universitat de Barcelona with Hospital Clínic de Barcelona and Ege Üniversitesi with Izmir University Medical Center. The Characteristics of the 6 countries’ educational contexts and nine selected clinical units where the DEU model was implemented are described in Table 1.

**Table 1:** Educational and clinical context characteristics

	Spain		Belgium				Poland	Portugal	Turkey
**EDUCATIONAL CONTEXT**	**University**		**University College**				**University**	**Polytechnic Institute**	**University**
	**Nursing degree 4 years 240 ECTS**		**Nursing degree** **3 years 210 ECTS**				**Midwife degree 3 years 210 ECTS**	**Nursing degree 4 years** **240 ECTS**	**Nursing degree 4 years** **240 ECTS**
**CLINICAL CONTEXT**	**General Hospital**		**General Hospital**		**General Hospital**		**Specialized maternity hospital**	**General Hospital**	**General Hospital**
**Number of beds**	**682**		**1756**		**811**		**297**	**376**	**2000**
**Number of staff Nurses/midwives**	**2305**		**2946**		**1250**		**216**	**695**	**58**
**Number of patients attended per year**	**40,451**		**693,592**		**36,090**		**19,255**	**170,000**	**1,000,000**
**Nurse or midwife/patient ratio in the unit**	**(unit A)**	**(unit B)**	**(unit A)**	**(unit B)**	**(unit C)**	**(unit D)**			
**Morning**	**1/8**	**1/2–3 (ICU)**^a^	**1/5–6**	**1/2–5 (ICU)**^a^	**1/9**	**1/9**	**1/11**	**1/4**	**1/70**
**Afternoon**	**1/8**	**1/2–3 (ICU)**^a^	**1/ 8–9**	**1/3–5 (ICU)**^a^	**1/9**	**1/9**	**1/11**	**1/6**	**1/25**
**Night**	**1/16**	**1/2–3 (ICU)**^a^	**1/ 20–61**	**1/4–5 (ICU)**^a^	**1/32**	**1/32**	**1/11**	**1/10**	**1/45**

^a^Intensive Care Unit

This study includes the perceptions of 21 key professionals from academic and service organizations who had an active role in DEU implementation: Belgium (*n* = 4), Poland (*n* = 5), Portugal (*n* = 5), Spain (*n* = 4) and Turkey (*n* = 4). Participants roles were: hospital and faculty managers and associate managers, faculty professors, and LT, responsible for coordinating DEU implementation and evaluation of each student’s CP. These professionals worked daily with various stakeholders, including clinicians, staff nurses-midwives, other health workers and the community, and this provides opportunities to deliver feedback during regular meetings. Assessing implementation from diverse perspectives ensured vital aspects (economic, organizational and human) were captured to contribute to fruitful implementation.

### Implementation strategy

A research team for each country was formed with a study coordinator designated for each team. The DEU unified guidelines for carrying out implementation and assessment were registered in a protocol [12]. Meetings with universities and healthcare directors were held, contracts and agreements were signed to arrange the educational-service partnerships and to adapt the DEU to each setting. Each organization selected the appropriate units based on students’ academic level, interest, motivation and the availability of nurses/midwives, or other institutional preferences. HN, LT and CM roles and responsibilities were identified, written into the implementation guidelines and given to all participants. The DEU mentoring course was imparted by experts to at least four members of each country’s research team. Afterwards, each member country was responsible for giving the course to HN, LT and CM project participants. This course provided global and specific per-country theoretical and practical knowledge, skills and strategies for dealing with potential issues arising during students’ CP. Staff from selected units were informed about the model and objectives. The first day of the students’ CP, trained CM and students were paired in one-on-one relationships and the LT was incorporated into the student and unit health team. Follow-up meetings and evaluation of the DEU implementation process and outcomes were conducted weekly and at the end of the program, respectively.

### Instrumentation/procedures for data collection

To evaluate DEU implementation, we used quantitative and qualitative measures, including structured and semi-structured interviews, focus groups, and observation with the aim of assessing organizational context attitudes and behaviors. In this part of the study, aiming to identify DEU implementation facilitators and barriers, we used the Consolidated Framework for Implementation Research (CFIR). It is considered that use of the CFIR may help to advance implementation science [13, 14]. The CFIR describes constructs related to the Process of implementation: Planning, Engaging, Executing, and Reflecting and Evaluating [15]. It is composed of five domains: intervention characteristics, outer setting, inner setting, characteristics of the individuals involved and process implementation. The CFIR has been applied in many studies and uses practical comprehensive taxonomy of constructs with the potential to influence implementation effectiveness and to encourage consistency in evaluation and reporting of translational efforts [16, 17]. We used the interview questions from the CFIR website [17] before and after the DEU implementation process. Likewise, we used the same authors’ criteria to qualify each item’s influence on implementation. For each construct, ratings were assigned that reflected the influence. Valences of + 2 + 1 reflect a positive influence, − 2 and − 1 a negative influence while 0 indicates that this construct had no influence on DEU implementation [16] (Table 2).

**Table 2:** Criteria used to assign ratings to constructs [16]. Authorized by Damschroder

Rating	Criteria
−2	The construct is a negative influence in the organization, an impeding influence in work processes, and/or an impeding influence in implementation efforts. The majority of interviewees (at least two) describe explicit examples of how the key or all aspects (or the absence) of a construct manifests itself in a negative way.
−1	The construct is a negative influence in the organization, an impeding influence in work processes, and/or an impeding influence in implementation efforts. Interviewees make general statements about the construct manifesting in a negative way but without concrete examples: (1) the construct is mentioned only in passing or at a high level without examples or evidence of actual, concrete descriptions of how that construct manifests; (2) there is a mixed effect of different aspects of the construct but with a general overall negative effect; (3) there is sufficient information to make an indirect inference about the generally negative influence and/or (4) judged as weakly negative by the absence of the construct.
0	A construct has neutral influence if: (1) it appears to have neutral effect (purely descriptive) or is only mentioned generically without valence; (2) there is no evidence of positive or negative influence; (3) credible or reliable interviewees contradict each other; (4) there are positive and negative influences at different levels in the organization that balance each other out; and/or different aspects of the construct have positive influence while others have negative influence and overall, the effect is neutral.
+ 1	The construct is a positive influence in the organization, a facilitating influence in work processes, and/or a facilitating influence in implementation efforts. Interviewees make general statements about the construct manifesting in a positive way but without concrete examples: (1) the construct is mentioned only in passing or at a high level without examples or evidence of actual, concrete descriptions of how that construct manifests; (2) there is a mixed effect of different aspects of the construct but with a general overall positive effect and/or (3) there is sufficient information to make an indirect inference about the generally positive influence.
+ 2	The construct is a positive influence in the organization, a facilitating influence in work processes, and/or a facilitating influence in implementation efforts. The majority of interviewees (at least two) describe explicit examples of how the key or all aspects of a construct manifests itself in a positive way.
_	Missing Interviewee(s) were not asked about the presence or influence of the construct; or if asked about a construct, their responses did not correspond to the intended construct and were instead coded to another construct. Interviewee(s) lack of knowledge about a construct does not necessarily indicate missing data and may instead indicate the absence of the construct.

#### CFIR domains and constructs definition

Intervention Characteristics: This domain refers to the main intervention attributes influencing the success of the implementation. Questions explore participants’ opinions about the individual or group carrying out the intervention, individuals’ participation in decision processes, previous information and solid evidence about the intervention, influential stakeholder support, degree of strength of intervention implementation, required changes, alterations and the cost of adapting the intervention to the organization.

Outer Setting: This domain relates to the current organizational situation requiring this intervention. Participants were asked about patients’ needs and preference awareness, how individuals are stimulated by the organization to take the initiative and make suggestions, the advantages of intervention implementation compared with similar organizations and external strategies to extend the intervention.

Inner Setting: This domain describes the organization’s characteristics. Questions are related to organizational structure and social design, the nature and quality of professionals’ communications, professionals’ incentives and remunerations, learning climate, and personal and economic resources.

Characteristics of individuals: This domain relates to individuals’ beliefs, attitudes and motivation to cope with changes, their self-perception with respect to the success of the implementation or their commitment to the organization.

Process: This domain relates to implementation planning in advance and the degree to which the plan is followed. Participants were asked about involvement of appropriate individuals and leaders, outside individuals who could influence and apply the implementation, and the nature and quality of participants’ feedback regarding planning, implementation and outcomes.

We examined rating patterns within and across organizations to identify barriers, facilitators and constructs that distinguished between settings. In addition, supplementary DEU-outcomes evaluation was added to analyse, quantitatively and qualitatively, nurses/midwives and students’ experiences and perceptions of DEU implementation and outcomes (Table 3).

**Table 3:** CFIR domains, constructs and supplementary evaluation

Consolidated framework for implementation research domains					Supplementary evaluation
Intervention characteristics	Outer setting	Inner setting	Characteristics of individuals	Process	
- Adaptability - Complexity - Cost - Design quality and packaging - Evidence strength and quality -Intervention source - Relative advantage -Trialability	- Cosmopolitanism - External policy and incentives - Patient needs and resources - Peer pressure	- Culture - Implementation climate (tension for change, goals & feedback, relative priority, compatibility, learning climate, organizational incentives & rewards) - Networks and communication - Readiness for implementation (available resources, leadership engagement, access to knowledge & information) - Structural characteristics	- Knowledge and beliefs about the intervention - Individual stage of change - Individual identification with the organization - Self-efficacy - Other personal attributes	- Engaging (champions, formally appointed implementation leaders, external change agents, opinion leaders) - Executing - Planning - Reflecting and evaluating	- Students and nurses/midwives focus groups. - CLES-T questionnaire for undergraduate students. - PES-NWI survey for nurses/midwifes. - Open-ended questions for nurses/midwives.

### Data analysis

Participants ratings for every CFIR construct were analized to determine if the construct had a positive, neutral, or negative influence on DEU implementation performance, and the degree of its influence. The five CFIR domains were used as a framework for identificable codes and constructs for categories. Two researchers read and re-read the participants’ answers and organized the data. The participants verified the data to ensure isomorphism between the data collected and reality and to maximize the validity of findings. Computer software (ATLAS-ti version 8.2.1) was used for exploration, management and evaluation of data. During the investigation, standards of quality and scientific rigor; credibility, transferability, dependence and reliability described by Lincoln and Guba (1985) were applied [18].

### Ethical considerations

The Ethics Committee at Hospital Clinic, Barcelona granted approval for this study (approval number: HCB/2017/0053). Staff received both written and oral information about the study aim and methodology. Written informed consent was signed before data collection and participants could withdraw from the study at any time. Participants’ data were kept confidential and their identifying information was removed and cannot be connected to them.

## Results

Although the DEU model implementation was heterogeneous, qualitative data from CFIR questions showed many more shared traits than differences in participants’ answers. Table 4 shows the characteristics of the nine DEU model units implemented. Table 5 shows the valences given by each group of coordinators to qualify each CFIR construct as having a positive, negative or neutral influence on DEU implementation. Results and participants’ illustrative quotations are presented as examples of the most common perceptions about facilitators and barriers in DEU planning and implementation.

**Table 4:** DEUs clinical learning environment

Deu clinical learning environment	Spain (unit A)	Spain (unit B)	Belgium (unit A)	Belgium (unit B)	Belgium (unit C)	Belgium (unit D)	Poland	Portugal	Turkey
CP duration in days.	32		45		35		22	42	35
CP duration in hours.	240		360		280		265	210	245
Number & year of students in DEU.	2 (4th year)	3 (4^th^year)	7 (3rd year)		6 (3rd year)		2 (1th year)	1 (3rd year) 1 (4th year)	3 (3rd year)
Trained CM in DEU during CP.	6		4		11		2	2	2
% Student-CM matched in one-to-one relationships	100%		85%		85%		100%	100%	100%
Days/week LT present in DEU during CP.	1 day/week		1 day/week		1 day/week		4 day/week	1 day/week	1 day/week
Hours/week LT present in DEU during CP.	6		8		8		32	1 or 2	8
DEU mentorship course duration in hours.	18		40+ 16^a^		40		40	12	18

^a^2 days/year of CM up-dating training

**Table 5:** CFIR constructs and ratings

CFIR domain	CFIR construct	Spain	Belgium (A&B)	Belgium (C&D)	Poland	Portugal	Turkey
**INTERVENTION CHARACTERISTICS**	Intervention Source	*+ 2*	*+ 1*	*+ 1*	*+ 1*	*+ 2*	*+ 1*
	Evidence Strength & Quality	*+ 2*	*+ 2*	*+ 2*	*+ 2*	*+ 2*	*+ 2*
	Relative Advantage	*+ 2*	*+ 1*	*+ 1*	*+ 1*	*+ 2*	*+ 1*
	Adaptability	*+ 2*	*+ 2*	*+ 2*	*+ 2*	*+ 2*	*+ 2*
	Trialability	*+ 2*	*+ 1*	*+ 2*	*+ 1*	*+ 2*	*+ 2*
	Complexity	*+ 2*	*+ 1*	*+ 1*	*+ 1*	*+ 1*	*+ 1*
	Design Quality & Packaging	*0*	*0*	*0*	*0*	*+ 2*	*0*
**OUTER SETTING**	Cost	*+ 1*	*+ 1*	*+ 1*	*+ 1*	*+ 1*	*+ 1*
	Patient Needs & Resources	*0*	*0*	*0*	*0*	*+ 2*	*0*
	Cosmopolitanism	*+ 2*	*+ 1*	*+ 1*	*+ 1*	*+ 2*	*+ 1*
	Peer Pressure	*+ 1*	*0*	*0*	*0*	*+ 1*	*0*
	External Policy & Incentives	*+ 1*	*+ 1*	*+ 1*	*+ 1*	*+ 1*	*+ 1*
**INNER SETTING**	Structural Characteristics	*+ 2*	*+ 1*	*+ 1*	*+ 1*	*+ 1*	*+ 1*
	Networks & Communications	*0*	*+ 1*	*+ 1*	*+ 1*	*+ 2*	*+ 1*
	Culture	*+ 1*	*+ 1*	*+ 2*	*+ 1*	*+ 2*	*+ 2*
	Implementation Climate	*+ 2*	*+ 2*	*+ 2*	*+ 2*	*+ 1*	*+ 2*
	Tension for Change	*0*	*+ 1*	*+ 1*	*+ 1*	*0*	*+ 1*
	Compatibility	*+ 1*	*+ 1*	*+ 1*	*+ 1*	*+ 2*	*+ 1*
	Relative Priority	*+ 1*	*+ 1*	*+ 1*	*+ 1*	*+ 1*	*+ 1*
	Organizational Incentives & Rewards	*+ 1*	*0*	*0*	*0*	*+ 1*	*0*
	Goals and Feedback	*+ 2*	*+ 2*	*+ 2*	*+ 2*	*+ 2*	*+ 2*
	Learning Climate	*+ 2*	*+ 2*	*+ 2*	*+ 2*	*+ 2*	*+ 2*
	Readiness for Implementation	*+ 1*	*+ 1*	*+ 1*	*+ 1*	*+ 1*	*+ 1*
	Leadership Engagement	*+ 2*	*+ 2*	*+ 2*	*+ 2*	*+ 2*	*+ 2*
	Available Resources	*+ 2*	*+ 2*	*+ 2*	*+ 2*	*+ 2*	*+ 2*
	Access to Knowledge & Information	*+ 1*	*+ 1*	*+ 1*	*+ 1*	*+ 2*	*+ 1*
**CHARACTERISTICS OF INDIVIDUALS**	Knowledge & Beliefs about the Intervention	*+ 2*	*+ 2*	*+ 2*	*+ 2*	*+ 2*	*+ 2*
	Self-efficacy	*+ 2*	*+ 2*	*+ 2*	*+ 2*	*+ 2*	*+ 2*
	Individual Stage of Change	*+ 1*	*+ 1*	*+ 1*	*+ 1*	*+ 2*	*+ 1*
	Individual Identification with Organization	*+ 1*	*+ 2*	*+ 2*	*+ 2*	*+ 2*	*+ 2*
	Other Personal Attributes	*+ 1*	*+ 1*	*+ 1*	*+ 1*	*+ 2*	*+ 1*
**PROCESS**	Planning	*+ 1*	*+ 2*	*+ 2*	*+ 2*	*+ 2*	*+ 2*
	Engaging	*+ 2*	*+ 2*	*+ 2*	*+ 2*	*+ 2*	*+ 2*
	Opinion Leaders	*+ 1*	*+ 2*	*+ 2*	*+ 2*	*+ 2*	*+ 2*
	Appointed Internal Implementation’ Leaders	*+ 2*	*+ 2*	*+ 2*	*+ 2*	*+ 1*	*+ 2*
	Champions	*+ 1*	*+ 1*	*+ 1*	*+ 1*	*+ 1*	*+ 1*
	External Change Agents	*+ 1*	*+ 1*	*+ 1*	*+ 1*	*+ 2*	*+ 1*
	Key Stakeholder	*+ 1*	*+ 1*	*+ 1*	*+ 1*	*+ 2*	*+ 1*
	Intervention participants	*+ 2*	*+ 2*	*+ 2*	*+ 2*	*+ 2*	*+ 2*
	Executing	*+ 1*	*+ 1*	*+ 1*	*+ 1*	*+ 2*	*+ 1*
	Reflecting & Evaluating	*+ 1*	*+ 2*	*+ 2*	*+ 2*	*+ 2*	*+ 2*

### Intervention characteristics

Project coordinators answered these domain questions before starting DEU model implementation. For these professionals, DEU model quality and adaptability were principal factors that positively influenced implementation. Spain participants added: *“Diverse literature describes the DEU model as an optimal context to contribute positively to the improvement of the capacities of the student and the balance of the nurses’ roles and to grow their professionalism.”*

Design and packaging were not perceived as influential since they considered that implementation protocols, guidelines and online resources were available, and that materials and support tools were consistently considered helpful by all participating centers. Likewise, training courses that were freely given to staff nurses/midwives by LTs helped CMs to carry out their teaching role. Furthermore, participants considered that the DEU was not a complex intervention, and every step could be planned in advance, so facilitating its implementation. Spain coordinators stated: *“Meetings between coordinators, nurses/midwives, head nurses/midwives, professors, lecturers and managers are planned with the aim of deciding on the changes we need to make and the resources we have to use to implement the intervention.”*

### Outer setting

Participants thought this domain had less positive impact on DEU implementation. In relation to patient needs and resources, all participants strongly agreed on the quality of the patient care guarantee because in the DEU model, at all times, students and patients were supervized by a qualified registered nurse/midwife. Participants in Belgium added: *“Because the students are always supervized by an experienced nurse, the quality of care could remain guaranteed. Because they were trained in total patient care, and because they were able to make time for the patient, it might even increase the ability to meet the needs.”* Turkish participants believed implementation of the DEU model in their units could increase the quality of nursing students’ education and the quality of patient care. They stated: “*They* (*patients*) *were happy about it because that intervention was to educate the nurses better and these nurses will attend them in the future.”* Further, coordinators in Portugal agree that *“The intervention will emphasize a closer approach to the assessment and follow-up of patients’ needs and preferences.”*

All project coordinators believed their organizations had higher quality networking, and the option to keep in contact with other organizations where the DEU is already implemented was an additional benefit at the time of collecting, sharing and comparing experiences. Belgium participants stated: *“We talk about the DEU with all other DEUs on a formal basis. To evaluate the DEU, but also to exchange experiences. We do this with other DEUs in the hospital, but also with other partners from other hospitals, even from other schools. Then, of course, also with our international partners.”* Spain participants stated*: “We promote transversality between different organizations and departments to promote team work and professional involvement.”*

Belgium coordinators stated that the transition of the Nursing degree from year 3 to year 4 and the increase of students’ CP hours was a performance measure that influenced DEU implementation because it implied changes, evaluation of current strategies and implementation of best practices: *“On the macro, meso and micro levels everybody is discussing the quality of internships because the impact is greater which means the quality needs to be high.”*

### Inner setting

Participants considered the inner setting the most positive influential CFIR domain at the time of DEU implementation. They highlight leadership engagement from the project’s coordinators and declared that the educational and health teams and management were open-minded, included innovations and engaged people at all levels. They also stressed the freedom of the learning climate in allowing people to demonstrate their capacities and creativity to improve any intervention or innovation. Portugal participants added: *“ The institution is an “open door hospital“, which values the partnership relationship with the various community structures, with established protocols with the city’s existing dynamism.*” and *“There is a creative freedom within the institutional rules on quality.”* Poland coordinators agree: *“Our university tries to be up-to-date with new methods, because they are very important in medicine.”*

Participants stated that the common highest incentive and reward in DEU implementation was the satisfaction that the DEU learning and work environment brings to both students and nurses/midwives. Belgium participants highlighted: *“Students who make positive development during the internship. Validation of the role of mentor and link teacher. Satisfying relationships with the participants. Mentors experiencing the power of their competences that results in stronger self-confidence as mentor; also a certificate of participation for team/mentors and students.”*

Through the utilization of the knowledge, skills and resources of both clinical and academic partners, participants could share and pool implementation strengths. Moreover, the opportunity to collect data on DEU implementation processes and outcomes was important in assessing DEU efficacy and possible benefits. Spain participants stated: *“Based on the project and through a data collection system, we will learn about the experience, opinions and suggestions of everyone involved: Mentors, Teachers, Head Nurses, students and project coordinators.”*

### Characteristics of individuals

All participants answered questions from this domain, identifying it as a facilitator in the DEU implementation process. Nurses/midwives considered themselves motivated and committed people and highly qualified professionals. Participants believed that self-efficacy and personal confidence in the health and educational team were decisive when implementing changes. Spain participants added: *“Confident, hopeful, courageous. I believe in the preparation, motivation and interest of the teams.”*

Also emphasized were the participants’ commitment to the organization, as well as the professionals’ perception that organizational values and culture are focused on students and professionals’ well-being and development. Other personal traits such as self-confidence, competence, capacity and aptitudes to undertake the project were mentioned by participants. Belgium participants stated: *“I consider that I present motivation, and ability to motivate others to change. In addition, I contribute, from the research team, to develop interventions to implement and pilot the change in such a way that it can be evaluated through the improvement of care.”*

### Process

Questions in this domain were answered after DEU model implementation. Engaging, Intervention participants and Reflecting & Evaluating constructs were considered by participants to be the most influential positive factors during the DEU implementation process. DEU coordinators and implementation teams’ effective leadership were strategic advantages for participants and helped to overcome difficulties in DEU implementation.

Portugal participants stated: *“Motivated and experienced ones (leaders), both in nursing care and student mentoring.”* They also added: *“Choose available and experienced CM. Project marketing at the unit. Adequate training and involvement in the project. Continuous assessment of the project.”*

Engaging and involving experienced and motivated people, training them to lead the implementation and to attract other professionals were beneficial to the success of the intervention. Spain coordinators declared: *“People believe that all those involved in the project have skills, and are motivated and prepared. Leaders, Coordinators, Mentors, Link Teachers, researchers and nurses who, in addition to being very prepared and motivated, spread their enthusiasm and interest to the rest of the teams.”*

The opportunity for weekly DEU model meetings to identify, evaluate and handle process and outcomes issues, and the open and continuous communication channels to stimulate feedback between all parties involved, were represented as facilitators of implementation success. Portugal participants added: *“The evaluation has been continuous. There have been weekly meetings with those involved and an open communication channel with all the participants all the time.”*

## Discussion

This is the first process evaluation of DEU implementation in 6 European undergraduate nursing/midwifery student CP settings and the first evaluation to use the CFIR. The use of CFIR implementation science theory allowed us to explore nurses/midwives’ perceptions on the factors hindering or enabling DEU implementation and to ‘unpack’ the reasons that professionals believed the intervention was implemented successfully. Participants did not find barriers across CFIR constructs although there were divergences with respect to the influence of constructs at the time of DEU planning and implementation.

Regarding respondents’ perception, “Intervention characteristics” was highlighted as an aid to the implementation process. DEU evidence-based quality was significant in planning, readiness and implementation processes. Organizations that support safety and quality in health care encourage DEU replication in numerous sites due to the reported benefits of the DEU for educational and healthcare organizations. Additionally, the availability of several protocols, guidelines and the literature on the DEU reduced implementation complexity, facilitated the proposed strategy and its adaptation to dissimilar sites [7–11, 19]. Our results are comparable with those which show that intervention adaptability seems to be guaranteed when educational-service collaboration is based on trust, respect and mutually beneficial goals: effective use of existing resources, support for professional improvement, nursing workforce recruitment and retention, and awareness of the teaching/learning process [20, 21].

Collaboration between educational and healthcare resources and their cooperation with other national or international organizations is reflected in the ‘Cosmopolitanism’ construct in the CFIR framework. Excellent organizational communication and networking are considered facilitators in DEU implementation. The organization’s expanded networks, together with DEU international expansion, could respond to international nursing education concerns about differences in nursing training requisites and experiences, represent a strategic opportunity to increase cooperation and mutual understanding, and facilitate nurses and students’ international mobility [6, 22, 23].

We found that the “Inner setting” domain displayed more positive influence in a successful DEU implementation. Our results are in line with those of Varsi et al. (2015) [24] that showed that their organizations’ structural characteristics, available resources, workplace culture and implementation climate influenced the implementation of innovations. The nursing work environment is characterized by constant change, and nursing staff are skilled in process and strategy modifications and adaptations [25]. Organizations’ readiness to change was based on the experience of implementation of innovations, professionals’ insight into organizational receptiveness, openness to new interventions, and encouragement of professionals to contribute ideas and opinions. Other authors also highlighted significant leadership engagement and available resources to implementation of changes [26–28].

We observed in the “Characteristics of Individuals” domain that the presence of positive perceptions of team involvement and willingness to collaborate were more likely to facilitate change at the time of implementing the DEU. Professionals’ perception of gratifying experience in the DEU was reported in several studies [7–9] and the impact on patient care and the health system of nurses’ confidence and satisfaction with their skills, knowledge and teaching role is shown [29]. As in the study by Glynn et al. [30], participants stated that effective communication between educational and healthcare organizations is necessary to provide nurses with educational role skills, role expectations and clarification. Likewise, it is known that sustaining this kind of educational-service collaboration is indispensable in supporting professionals, reinforcing new organizational structures and offering recognition and reward for all parties involved [30, 31].

Answers related to the “Process” domain showed that shared participants’ expectations, suggestions and questions during meetings and training courses brought greater opportunities to make adjustments and adapt the DEU to each context. Professional training and ongoing opportunities to learn how to teach students helped to improve professionals’ self-confidence in their competences. It is a recommendation for improving nursing education and staff nurses’ demands in the workplace [30, 31]. Moreover, the establishment of a safe space for professional discussion and satisfaction was achieved when their feedback and suggestions were accepted and seen as factors that create a positive clinical-learning environment [32].

We found the CFIR to be useful and practical tool for analyzing DEU implementation success determinants. Data about implementation processes and outcomes were important for assessing efficacy and possible benefits, and encourage reflection and team discussion aiming to detect implementation difficulties and seek solutions. The identification, supervision and handling of process and outcome data represented an aid to implementation success. The CFIR identified factors that could influence the success of implementation when moving an evidence-based intervention to a new setting [24]. In addition to helping users understand what works or does not work in implementation research, the qualitative-based CFIR also helps researchers understand how and why implementation processes work [15, 17]. In addition, our mixed-method supplementary evaluation (nurses/midwives and students’ focus groups and questionnaires) created a rich pool of qualitative and quantitative data to meet the research aim. Results from supplementary evaluation will be reported in a further paper.

## Limitations

Our study is limited to a small number of educational and healthcare organizations per country as it was a DEU pilot implementation in each country. Another limitation can be found in the singular elements of different curricula of educational institutions and different health care systems of healthcare providers participating in this study, which we have not subjected to a deep analysis. Therefore, its results are not representative of other European contexts, for instance, community hospitals. Although, in order to avoid possible language barriers in the collection of qualitative data, the research staff were trained to provide interpreter or translator services to translate the participants’ data into the English language, this may be a limitation in a qualitative cross-language study.

## Conclusion

Despite the limitations, this study gives us a complete picture of how DEU model implementation and outcomes were considered in practice. The authors can confirm that these selected methods were appropriate to the research aims of this implementation evaluation. We believe this qualitative process evaluation were interested and keen to contribute to the process analysis, and created a rich pool of data.

This study facilitates the key elements to guide educational and healthcare stakeholders in DEU implementation. It may help educational and healthcare organizations to engage people in the whole process and allow other organizations to consider DEU model benefits and sustainability.

The CFIR was able to outline those organizational, individual behavior and external agency factors that have a direct impact on DEU implementation. Use of the CFIR to guide and evaluate intervention and implementation allows researchers to compare their findings with other studies and to promote discussion about future research.

## Implication for practice

This article highlights the practical benefits for nurse managers and researchers when translating research findings into practice and contributes strategies that organization leaders could explore prior to implementing the DEU model in healthcare settings. Educational and health care managers can draw on the five CFIR framework domains and consider them in the routine of change or innovations implementation and outcomes evaluation. Additionally, our findings could inform future efforts by helping to explain why implementation went well or not.

## Data Availability

The datasets generated and/or analyzed during the current study are not publicly available, but are available from the corresponding author on reasonable request.

## Abbreviations

DEU: Dedicated education units
CFIR: The Consolidated Framework for Implementation Research
CP: Clinical placement
CM: Clinical mentor
LT: Link teacher
HN: Head nurse
